# Dysbiosis: A Potential Precursor to the Development of a Depressive Disorder

**DOI:** 10.3390/healthcare10081503

**Published:** 2022-08-10

**Authors:** Seung-Young Chung, Karel Kostev, Christian Tanislav

**Affiliations:** 1 GP Practice BAG Chung and Wiens, 66538 Neunkirchen, Germany; 2 Epidemiology, IQVIA, 65901 Frankfurt am Main, Germany; 3 Department of Geriatrics and Neurology, Diakonie Hospital Jung Stilling Siegen, 57074 Siegen, Germany

**Keywords:** dysbiosis, depression, microbiome, gut flora, mood disorder

## Abstract

Background: Although previous investigations have indicated that gastrointestinal pathologies facilitate the occurrence of mood disorders, there is a lack of studies based on data from clinical practice. The aim of this study was to investigate the incidence of depression in patients with dysbiosis. Methods: Adult patients (≥18 years) from 1193 general practices in Germany between January 2005 and December 2018 with an initial diagnosis of dysbiosis documented anonymously in the Disease Analyzer database (IQVIA) were analyzed. The incidence of depression diagnoses as a function of dysbiosis was calculated and multivariate regression models were applied. Results: This study included 552 patients with and 552 patients without dysbiosis. Within five years of the index date, 20.5% of patients with dysbiosis and 5.5% of individuals without dysbiosis had been diagnosed with depression (*p* < 0.001). Dysbiosis was found to be significantly associated with the incidence of depression (HR: 2.85 (95% CI: 2.00–4.04)). This association was slightly stronger in men (HR: 3.54) than in women (HR: 2.61) and was more pronounced in the age group >60 years (HR: 4.43). Conclusions: We identified dysbiosis as a risk factor for developing depression within 5 years after the index date. This risk seems to be higher in male than in female patients.

## 1. Introduction

Over the last decade, the relevance of imbalances in the microflora of the human gastrointestinal tract to the pathogenesis of various diseases has increasingly become the focus of medical research [1,2,3]. The qualitative and quantitative microbial miscolonization of the intestine may result not only in intestinal diseases, but could also lead to various other diseases of different types [4,5]. The potential pathophysiological relevance of dysbiosis has been the subject of many studies from various medical disciplines, rendering findings that indicate a causative implication [1,2,3]. Han and colleagues described a 3.4-fold increase in the hazard ratio in patients with dysbiosis for developing non-alcoholic fatty liver disease [6]. In a recent publication, Fields et al. speculated that there was a relationship between dysbiosis and the pathophysiology of neurodegenerative disorders such as Parkinson’s disease [7]. Increasing knowledge about the pathophysiological implication of dysbiosis has allowed new therapeutic approaches to be developed [4,8]. Intriguing data from South Korea indicate a lower prevalence of multiple sclerosis in individuals following a probiotic diet [4]. However, as well as somatic diseases that seem to be linked to enteral dysbiosis, there are also psychiatric disorders [9]. The microfloral pattern in the gut seems to have an influence on the cerebral metabolism [10].

Several previous investigations indicate that gastrointestinal pathologies facilitate the occurrence of mood disorders [11,12,13,14,15,16].

Although these previous studies have advanced the field, most were conducted in Asia and their findings may not be generalizable to countries in Europe. In addition, these studies often included small sample sizes and heterogeneous methodologies, or used animal data. In this context, further research is warranted based on data from clinical practice focusing on the relationship between dysbiosis and depression.

Therefore, we aimed to study the incidence of depression in patients with enteral dysbiosis, using data from a large database supplied by general practitioners in Germany.

## 2. Materials and Methods

### 2.1. Database

This study used data from the Disease Analyzer database [IQVIA]. Full details of the database have been published elsewhere [17]. This database contains demographic, diagnosis, and prescription data from general and specialized practices in Germany and has a coverage of approximately 3% [17].

### 2.2. Study Population

**Figure 1:**
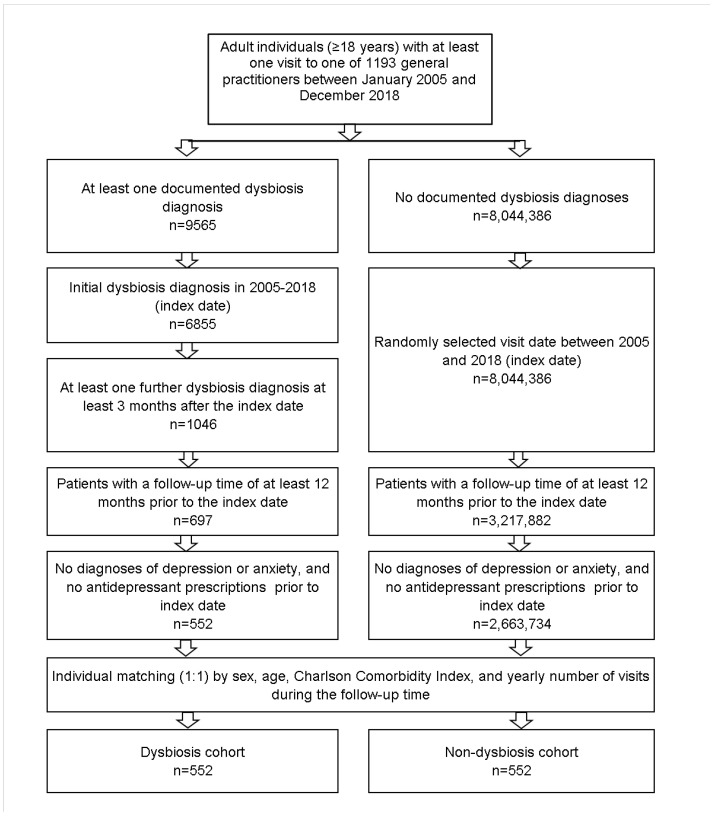
Selection of study patients.

This retrospective cohort study included adult patients [≥18 years] with an initial diagnosis of dysbiosis found in the original diagnosis text, from 1193 general practices in Germany between January 2005 and December 2018 [index date; Figure 1]. Patients were included if they had a further dysbiosis diagnosis three months or later after the index date, as this would indicate a chronic disease. A further inclusion criterion was an observation time of at least 12 months prior to the index date. Patients with depression [ICD-10: F32, F33], anxiety disorder diagnoses [ICD-10: F41], or antidepressant prescription [ATC: N06A] prior to the index date were excluded.

All individuals with at least one GP consultation between January 2005 and December 2018, with an observation time of at least 12 months prior to this consultation but without dysbiosis diagnosis their history, were included as the non-dysbiosis cohort.

Dysbiosis patients were propensity-score-matched to patients without dysbiosis by age, sex, Charlson comorbidity score, and yearly number of visits during the follow-up time. The Charlson index is a weighted index that accounts for the number and severity of comorbidities in administrative database studies, and includes a wide range of comorbidities [macrovascular diseases, pulmonary diseases, gastrointestinal, liver, and renal diseases, diabetes, tumors, and AIDS] [18]. In cases where several non-dysbiosis patients had the same propensity scores to be matched, a matched pair was randomly selected. For patients without dysbiosis, the index date was that of a randomly selected visit between January 2005 and December 2018 [Figure 1].

### 2.3. Study Outcomes and Statistical Analyses

The main outcome of the study was the incidence of depression diagnoses as a function of dysbiosis. We also analyzed the incidence of antidepressant therapy, defined as the prescription of an antidepressant drug following the diagnosis of depression.

The cumulative incidence of depression and antidepressant therapy in the dysbiosis and non-dysbiosis cohorts was calculated for up to five years after the index date, using Kaplan–Meier curves. Patient data were anonymized at the time of first depression diagnosis or on the day of the last database record of each individual patient, whichever occurred first. As no information on death was available, dead patients were considered lost to follow-up in this study.

Cox regression models were used to study the association between dysbiosis and depression, as well as antidepressant therapy incidence. These models were applied separately for four age groups [18–40, 41–50, 51–60, >60] and for women and men. *p*-values < 0.05 were considered statistically significant. Analyses were carried out using SAS version 9.4 [SAS Institute, Cary, NC, USA].

## 3. Results

### 3.1. Basic Characteristics of the Study Sample

The present study included 552 patients with dysbiosis and 552 patients without dysbiosis. The basic characteristics of study patients are displayed in Table 1. The mean age was 49.6 years [SD: 8.2 years]; 61.4% were women. The mean CCI was 1.5 [SD: 1.8] in both cohorts, without any significant difference.

### 3.2. Association between Dysbiosis and Depression

**Figure 2:**
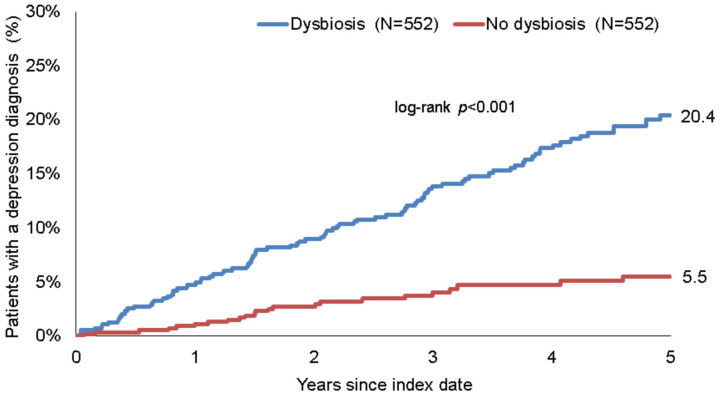
Kaplan–Meier curves for time to depression diagnosis in patients with and without dysbiosis.

Within five years of the index date, 20.5% of patients with dysbiosis and 5.5% of individuals without dysbiosis had been diagnosed with depression [log-rank *p* < 0.001] [Figure 2].

In the regression analyses, dysbiosis was found to be significantly associated with the incidence of depression [HR: 2.85 [95% CI: 2.00–4.04]]. This association was slightly stronger in men than in women, and was at its strongest in the age group >60 years [Table 2].

### 3.3. Association between Dysbiosis and Prescription of Antidepressant Drugs

**Figure 3:**
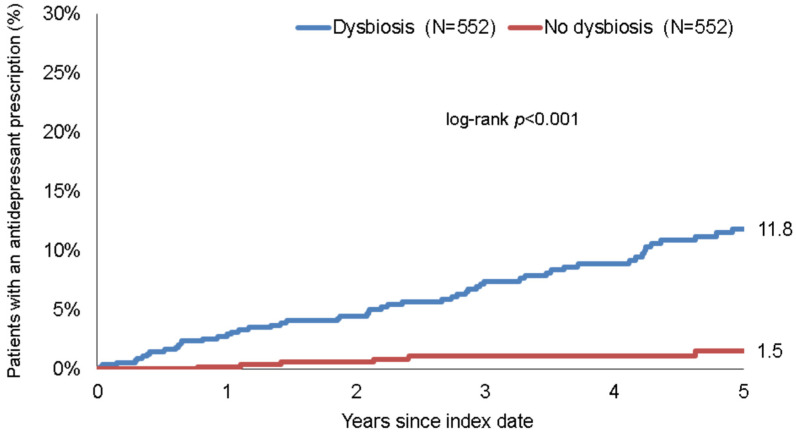
Kaplan–Meier curves for time to the first prescription of an antidepressant drug in patients with and without dysbiosis.

Within five years of the index date, 11.8% of dysbiosis patients and 1.5% of non-dysbiosis patients had received a prescription for antidepressants following a depression diagnosis [*p* < 0.001] [Figure 3].

In the regression analyses, dysbiosis was found to be significantly associated with the prescription of antidepressant drugs [HR: 4.12 [95% CI: 2.35–7.21]]. This association was stronger in women than in men. A very strong association [OR = 10.59] was observed in patients aged >60 years [Table 2].

## 4. Discussion

We identified the diagnosis of enteral dysbiosis as a precursor to developing a depressive disorder. Our study indicated that patients diagnosed with dysbiosis had a 20.5% relative risk of developing depression within five years after the index diagnosis. A 2.85-fold higher risk of depression was calculated for patients diagnosed with dysbiosis, compared with non-dysbiosis controls. This effect was especially pronounced in the age categories 18–40 years [HR: 3.77] and >60 years [HR: 4.43], respectively. We found a higher hazard ratio with regard to the risk of being diagnosed with depression within five years in male dysbiosis patients than in female dysbiosis patients [HR: 3.54 versus HR: 2.61]. In line with this finding, we also observed higher prescription rates for antidepressants in patients with dysbiosis [dysbiosis: 11.8% versus 1.5%].

In the literature, an imbalance of gut microbiome has been described as having an influence on various brain functions and thereby interfering with learning ability, behavior, anxiety, and mood [19]. While the pathophysiology behind this phenomenon is complex, it has been postulated that a role is played by disturbances in what is known as the gut–brain axis. Among several suspected mechanisms, modulation triggered by microbial metabolic products affects neurotransmitters and various processes via the vagus nerve, and microbial immunomodulation can cause neuroinflammation, with obvious impacts on behavior and mood [20]. Therefore, it appears likely that depressive symptoms would occur frequently in patients with an imbalance of intestinal flora. The increased frequency of depression among patients with gastrointestinal disorders appears to support this hypothesis [21,22,23]. Studies have shown that depressive symptoms occur in up to 20–40% of patients with chronic inflammatory bowel disease [21,22]. In patients with irritable bowel syndrome, the prevalence of depression was found to be around 25% [22]. However, the research to date has not tended to focus on the relevance of isolated dysbiosis without an underlying somatic disorder. Nevertheless, it is possible indirectly to draw some conclusions on this topic; investigations have identified positive effects on mood disorders achieved by influencing the microbiome through nutrition and the intake of microbiota [19,23,24]. On the other hand, different microbiome patterns might be associated with the occurrence of depressive symptoms [14]. To the best of our knowledge, our study is the first investigation to analyze the relevance of dysbiosis with respect to depression in individuals without underlying disease of the gastrointestinal tract. In our study, the 20.5% rate of depression detected in patients with dysbiosis seems to be in the same range as that for patients with other disorders of the gastrointestinal tract. We also identified dysbiosis as a risk factor for the development of depressive symptoms within five years of diagnosis.

Our study found differences in sex distribution as well as hazard ratios with regard to developing depression after diagnosis of dysbiosis. In our group of patients with dysbiosis [*n* = 552], 61.4% were female and 38.6% were male. It is possible that this observation could have been influenced by selection bias, but it can also be speculated that this result was specifically sex-determined, since sex hormones seem to have an influence on gut flora [25]. In a recent publication, Thackray and colleagues described a hormone-dependent change in the microbiome pattern in female patients with polycystic ovary syndrome, indicating that females are more likely to experience dysbiosis [25]. In contrast to this result, we detected a higher hazard ratio with respect to the odds of developing a depressive disorder in male versus female dysbiosis patients [HR 3.54 versus 2.61]. It can reasonably be assumed that there is a mutual relationship between antidepressant prescription and the occurrence of depression; the likelihood of subsequent depression in female dysbiosis patients was lower than that in male patients. In a 2006 paper, Pinto-Meza et al. reported positive effects of and an increased adherence to selective serotonin reuptake inhibitors [SSRIs] in postmenopausal women [26]. Therefore, it can be speculated that some of the female patients in our study group may have been prescribed antidepressants for other reasons, masking any subsequent depression disorder. However, further investigations regarding sex-related differences in dysbiosis and their relevance for the occurrence of a depressive disorder are needed to clarify interactions between different factors.

This study was subject to several limitations. First, diagnoses were analyzed solely on the basis of ICD-10 codes, and no data were available on the diagnosis process or severity of the disease. In addition, dysbiosis diagnoses are likely to have been underestimated, as this condition is only rarely tested in primary care settings. Second, because information on behavioral factors [e.g., alcohol use, smoking, and sedentary lifestyle] was lacking, the roles played by these factors could not be studied. Finally, due to the retrospective nature of the analysis, no causal relationships could be reported; instead, we focused solely on associations. However, the database provided real-world data from clinical practice.

## 5. Conclusions

Our study identified dysbiosis as a risk factor for developing depression within five years after the index diagnosis. Based on our findings, it can be assumed that patients with dysbiosis have a 2.85-fold higher risk of a subsequent diagnosis of depression. This risk seems to be slightly higher in male dysbiosis patients. Although our study was subject to several limitations, it represents the first attempt at estimating the risk of diagnosis with a depressive disorder following diagnosis of dysbiosis.

Based on the findings of this study, mental health should be assessed shortly after the initial diagnosis of dysbiosis, and also on a regular basis in the five years following the diagnosis. In terms of future research, more prospective data are needed to investigate the association between dysbiosis and depression, and to better characterize the factors playing a mediating role in this association.

## Figures and Tables

**Table 1:** Basic characteristics of the study sample (after 1:1 propensity score matching by sex, age, Charlson comorbidity index, and yearly number of visits during follow-up).

Variable	Proportion Affected among Patients with Dysbiosis (%) *n* = 552	Proportion Affected among Patients without Dysbiosis (%) *n* = 552	*p*-Value
Age (Mean, SD)	49.6 (18.2)	49.6 (18.2)	0.990
Age 18–40	32.8	33.7	0.808
Age 41–50	19.3	17.4	
Age 51–60	18.3	20.7	
Age >60	28.3	29.5	
Sex			
Female	61.4	61.4	1.000
Male	38.6	38.6	
Yearly number of visits during the follow-up	1.5 (1.8)	1.5 (1.8)	1.000
Charlson Comorbidity Index (Mean, SD)	5.0 (4.9)	5.0 (4.9)	0.970

Proportions of patients are given in in % unless otherwise indicated. SD: standard deviation.

**Table 2:** Association between dysbiosis and the incidences of depression and antidepressant therapy in patients followed in general practices in Germany (Cox regression models).

	Depression		Prescription of Antidepressants	
Variable	HR (95% CI)	*p*-Value	HR (95% CI)	*p*-Value
Total	2.85 (2.00–4.04)	<0.001	4.12 (2.35–7.21)	<0.001
Age 18–40	3.77 (1.88–7.56)	<0.001	4.61 (1.57–13.50)	0.005
Age 41–50	2.04 (0.94–4.42)	0.073	2.08 (0.65–6.67)	0.218
Age 51–60	1.93 (1.01–3.66)	0.045	2.80 (1.00–7.83)	0.049
Age >60	4.43 (2.06–9.53)	<0.001	10.59 (2.49–44.98)	0.001
Sex				
Female	2.61 (1.74–3.92)	<0.001	4.55 (2.31–8.99)	<0.001
Male	3.54 (1.75–7.14)	<0.001	3.09 (1.14–8.40)	0.027

## Data Availability

Derived data supporting the findings of this study are available from the corresponding author [Karel Kostev] on reasonable request.
